# Parentage assignment with genomic markers: a major advance for understanding and exploiting genetic variation of quantitative traits in farmed aquatic animals

**DOI:** 10.3389/fgene.2014.00432

**Published:** 2014-12-12

**Authors:** Marc Vandeputte, Pierrick Haffray

**Affiliations:** ^1^INRA UMR1313 Génétique Animale et Biologie Intégrative, Institut National de la Recherche Agronomique, Jouy en Josas, France; ^2^Ifremer, Institut Français de Recherche pour l’Exploitation de la Mer, Palavas-les-Flots, France; ^3^Sysaaf, Syndicat des Sélectionneurs Avicoles et Aquacoles Français, Rennes, France

**Keywords:** aquaculture, parentage assignment, selective breeding, microsatellites, SNPs

## Abstract

Since the middle of the 1990s, parentage assignment using microsatellite markers has been introduced as a tool in aquaculture breeding. It now allows close to 100% assignment success, and offered new ways to develop aquaculture breeding using mixed family designs in commercial conditions. Its main achievements are the knowledge and control of family representation and inbreeding, especially in mass spawning species, above all the capacity to estimate reliable genetic parameters in any species and rearing system with no prior investment in structures, and the development of new breeding programs in many species. Parentage assignment should not be seen as a way to replace physical tagging, but as a new way to conceive breeding programs, which have to be optimized with its specific constraints, one of the most important being to well define the number of individuals to genotype to limit costs, maximize genetic gain while minimizing inbreeding. The recent possible shift to (for the moment) more costly single nucleotide polymorphism markers should benefit from future developments in genomics and marker-assisted selection to combine parentage assignment and indirect prediction of breeding values.

## INTRODUCTION

Aquaculture is now the fastest growing animal production worldwide, and provides half of the fish for human consumption worldwide (FAO, 2014). Such an important sector would be expected to use the best knowledge-based improvement methods, amongst which selective breeding is of paramount importance. However, Gjedrem et al. (2012) estimated that only 10% of aquaculture production worldwide is based on genetically improved stocks. There may be several reasons for this, but one clear technical weakness of aquaculture regarding the development of optimized selective breeding schemes is the fact that pedigree information is difficult and costly to obtain.

The basic reason is rather straightforward: farmed aquatic animals are all too small at hatching (from a few micrograms in mollusks and crustaceans to ca. 100 mg in salmonids fishes) to be physically tagged.

Then, there were initially two ways for fish genetic studies and breeding programs to deal with the question of pedigrees. The first and simpler solution was not to use a pedigree, using individual selection. In this case, fish are selected solely based on their own individual phenotype (see review in Gjedrem and Thodesen, 2005). Although effective to obtain genetic gain, this method is very limiting for studying genetic variation as: (1) it provides results only after a minimum of two generations, (2) it requires the maintenance of at least two fish lines, selected/control or divergent lines, (3) it limits the evaluation of genetic variation to one trait only, and (4) the precision of realized heritability estimates is low in reasonably-sized two generation experiments (Nicholas, 1980).

The second option to solve the pedigree problem is to use separate rearing of families until a size where tagging is possible, as in the Norwegian salmon breeding program, the first family-based selective breeding program in aquaculture, started in 1972 (Gjedrem, 2010). This was successfully extended to major aquaculture species such as salmonids, tilapias, oyster, or shrimps (Krishna et al., 2011; Thodesen et al., 2011; Gjedrem, 2012; Gjedrem et al., 2012; Zak et al., 2014). Although efficient, this method has three main drawbacks when it comes to estimating genetic parameters of traits. First, as families are reared separately, common environmental effects between tanks may inflate heritability estimates. The second point is that studying genetic variation with separate rearing of progenies requires the pre-existence of the family rearing units–i.e., of the infrastructure of the breeding program. Exploratory studies are then difficult to undertake. The third point is that the number of families is limited to the number of family rearing units used. Then, mating designs are constrained to those where the number of families produced is low for a given number of parents tested, like single pair mating or nested designs, which, unlike factorial designs, do not allow the separation of additive, maternal, common environment, and dominance effects (Becker, 1967).

Therefore, the provision of a method to trace pedigrees in groups of mixed families, with any type of family structure, was expected to be of great interest to study genetic variation of quantitative traits in aquaculture species, and subsequently to set up new types of breeding programs.

The principles of parentage assignment were set up for livestock paternity testing with allozymes (Jamieson, 1965). In fish, the very first trials were done in the 1970s in Israel, also using allozymes, but the number of families that could be discriminated was very low (<10) and the use of the method was limited to carp in one research team (e.g., Brody et al., 1981). The real start of parentage assignment studies in fish was in the 1990s with the availability of microsatellite markers (Herbinger et al., 1995; Estoup et al., 1998).

## TECHNICAL ASPECTS OF PARENTAGE ASSIGNMENT

### PARENTAGE ASSIGNMENT METHODS

Basically, two computation methods are used for parentage assignment, exclusion-based methods and likelihood-based methods (see Jones et al., 2010 for a review). Exclusion is very simple and makes no hypotheses other than Mendelian segregation of alleles, but is very sensitive to genotyping errors. When error rates are moderate and theoretical assignment power is high, however, genotyping errors can be dealt with by allowing a limited number of allelic mismatches between an offspring and its parents alleles (Vandeputte et al., 2006), and exclusion remains the gold standard of parentage assignment (Yue and Xia, 2014). Exclusion programs used in aquaculture are PROBMAX (Danzmann, 1997), VITASSIGN (Vandeputte et al., 2006), and FAP (Taggart, 2007). Likelihood methods use a different approach, with probabilities. In this case, the most likely couple is chosen as the true one (eventually integrating a genotyping error rate), but the decision rules rely on hypotheses on allelic frequencies. Likelihood methods generally give higher assignment rates than exclusion with low power marker sets, but sometimes give inconsistent results (Herlin et al., 2007; Trong et al., 2013). Using sibship information in calculations can greatly improve the efficiency of the likelihood methods (Wang and Santure, 2009). Likelihood programs used in aquaculture are CERVUS (Kalinowski et al., 2007), PAPA (Duchesne et al., 2002), and PARENTE (Cercueil et al., 2002).

A specific question is also the assignment of polyploids, especially sturgeons (Rodzen et al., 2004) or induced polyploids (Miller et al., 2014), and specific packages have been developed for tetraploids (wHDP; Galli et al., 2011), diploids to octoploids (VITASSIGN-OCTO; Vandeputte, unpublished), as well as a general method to transform polyploid genotypes to pseudo-diploid dominant genotypes (Wang and Scribner, 2014).

### A CRUCIAL ISSUE: THE ASSIGNMENT POWER OF MARKERS USED

However, whatever the method used, the first requirement to be able to use parentage assignment in practice is to obtain high levels of unique assignments, which primarily depends on the assignment power of the marker set used. It depends on the exclusion probabilities of the markers used and on the size of the problem to be solved, the total number of putative parents having an exponential effect on the proportion of unassigned individuals (Vandeputte, 2012). Overestimation of the assignment power of markers is very frequent (Vandeputte et al., 2011), and can be explained by Hardy–Weinberg disequilibrium (Wang, 2007), sampling variance and relatedness of parents (Villanueva et al., 2002; Matson et al., 2008), incomplete genotypes, genotyping errors especially caused by stuttering or size-shift (Sutton et al., 2011; Yue and Xia, 2014), and null alleles (Christie, 2010). In some species groups like mollusks, null alleles may be extremely frequent and problematic (Hedgecock et al., 2004), but the main cause of overestimation of the theoretical assignment power is a widespread inappropriate calculation method (Vandeputte, 2012). Typically, assignment power >0.99 can generally be obtained by 8–15 microsatellite markers in fish crosses involving a few tens or hundreds of parents, and a reasonable option when designing a marker set is to include a few more markers than theoretically needed. This then spares a lot of time by providing easy assignment even if small problems of genotyping errors, inbreeding or null alleles appear. High quality genotyping is also essential, and a recent review by Yue and Xia (2014) gives very useful insights to this question.

### MICROSATELLITES AND SNPS FOR PARENTAGE ASSIGNMENT

Microsatellites, due to their high number and high variability, are the markers that allowed the development of efficient parentage assignment methods. Today, however, SNPs (single nucleotide polymorphisms) use is growing exponentially (Guichoux et al., 2011), but not yet in parentage assignment. It was estimated that ∼6 SNPs give the same assignment power as 1 microsatellite (Glaubitz et al., 2003). Empirical studies tend to suggest that the adequate number of SNP for an efficient panel would be in the 100–450 range (Trong et al., 2013; Lapègue et al., 2014; Nguyen et al., 2014; Sellars et al., 2014). With such numbers, the classical requirement of unlinked markers within a panel cannot be met, thus lowering the real assignment power. SNPs are individually less expensive to genotype than microsatellites, but multiplexing decreased the cost of microsatellites genotyping (Guichoux et al., 2011; Yue and Xia, 2014), and for the moment SNPs remain more expensive due to the number required, but technology is rapidly evolving for SNPs and not for microsatellites. Empirical studies also sometimes reveal quite a high number of genotyping error in SNPs (Trong et al., 2013) and the necessity to test a higher number of SNP markers than expected to select the appropriate ones (Lapègue et al., 2014; Nguyen et al., 2014). However, prospects for development of genomic selection with low-marker density may imply genotyping of a few hundred to several thousand SNPs per fish (Lillehammer et al., 2013), which in this case would be sufficient to provide parentage assignment at no additional cost. The recent shift to SNP markers was, however, efficient to improve assignment at least in some mollusks species which suffered from high numbers of null alleles with microsatellites (Lapègue et al., 2014; Nguyen et al., 2014).

## IMPLEMENTATION OF PARENTAGE ASSIGNMENT IN AQUACULTURE

### INBREEDING CONTROL

Mass selection is the simplest way to improve traits such as growth or morphology, but bears a high risk of rapid genetic loss, with highly unbalanced families, which was revealed by parentage assignment mostly in mass spawning species (Perez-Enriquez et al., 1999; Waldbieser and Wolters, 1999; Boudry et al., 2002; Brown et al., 2005; Fessehaye et al., 2006; Herlin et al., 2007; Wang et al., 2008), but also in controlled artificial reproduction systems (Saillant et al., 2002; Kaspar et al., 2008).

The impact of different factors (mating design, mating ratio, number of parents per generation, selection pressure, trait heritability, grading practices) were simulated to improve inbreeding control and optimize genetic progress (Gjerde et al., 1996; Dupont-Nivet et al., 2006; Loughnan et al., 2013; Domingos et al., 2014), including optimal contribution selection, which requires pedigree knowledge (Sonesson, 2005; Skaarud et al., 2011).

### ESTIMATION OF GENETIC PARAMETERS

Estimation of heritability and genetic correlations allows to evaluate expected genetic gains and to design breeding programs. This is maybe where the possibility to access pedigree information by genotyping gave the most important and fruitful contribution to date to aquaculture genetics.

Optimization of mixed family designs for genetic parameters estimation was done by Vandeputte et al. (2001) for strain effects, Dupont-Nivet et al. (2002) for heritability, and Sae-Lim et al. (2010) for genotype by environment (G × E) interaction. After several feasibility studies with few families (Herbinger et al., 1995, 1999; Saillant et al., 2002), heritabilities were estimated in a growing number of fishes species for growth (Wilson et al., 2003; Saillant et al., 2006; Dupont-Nivet et al., 2008; Pierce et al., 2008; Wang et al., 2008; Gheyas et al., 2009; Domingos et al., 2013; Whatmore et al., 2013), processing traits (Kocour et al., 2007; Navarro et al., 2009; Saillant et al., 2009; Haffray et al., 2012a), flesh color (Norris and Cunningham, 2004), muscle fiber diameter (Vieira et al., 2007), deformities (Bardon et al., 2009), disease resistance (Guy et al., 2006; Antonello et al., 2009) or sex ratio (Vandeputte et al., 2007), and in shrimps and mollusks for growth (Jerry et al., 2006; Lucas et al., 2006; Kong et al., 2013; Nguyen et al., 2014) or meat yield in mussel (Nguyen et al., 2014). Heritabilities obtained in mixed family rearing are often higher than those recorded in separate rearing, which may be linked to the general absence of between-family environmental variance due to family mixing, although non-genetic maternal effects may persist in mixed family rearing (Haffray et al., 2012b), with possible upward biases on heritability estimates. Explicit comparisons of the same families in mixed or separate rearing design concluded that separate family rearing induced much higher levels of between-families environmental effects (Herbinger et al., 1999; Ninh et al., 2011).

Mixed family rearing also allowed the estimation of G × E interactions between rearing systems (Dupont-Nivet et al., 2008, 2010c; Navarro et al., 2009; Kvingedal et al., 2010; Domingos et al., 2013; Sae-Lim et al., 2013; Vandeputte et al., 2014), density or rearing temperatures (Saillant et al., 2006), plant-based vs marine feeds (Pierce et al., 2008; Le Boucher et al., 2013; Bestin et al., 2014), and even separated vs mixed fish family rearing designs (Ninh et al., 2011).

A limiting factor of such studies is that as fish are generally tagged to maximize individual information collection, individual performances are not available before physical tagging, thus limiting genetic studies on early stages. However, recent advances allow individual tagging at 200–400 mg (Ferrari et al., 2014), which should change this matter of fact.

### IMPLEMENTATION OF BREEDING PROGRAMS

#### Concepts used and implementation

The first proposal to use parentage assignment in breeding at an acceptable cost was an improvement of within-family selection called “walk-back selection” (Doyle and Herbinger, 1995). A two-step process of assignment was suggested and tested to achieve a minimal number of selected candidates per family (Herbinger et al., 1995).

**FIGURE 1:**
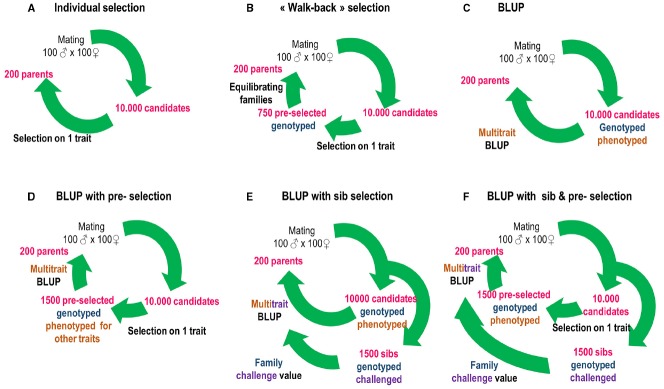
**Possible uses of genotyping to evolve from individual selection to BLUP (best linear unbiased prediction) and/or sib selection in aquaculture breeding.** Numbers are examples and have to be adapted for different species, traits, and breeding designs. **(A)** Under individual selection candidates are selected based on their own performance. **(B)** Under walk-back selection, animals selected on their individual performance are genotyped, and a subset of those with balanced family representation is used as broodstock. **(C)** Under BLUP, all animals are genotyped and phenotyped (possibly for several traits) and selected based on a BLUP breeding value combining phenotypic and pedigree information. **(D)** BLUP with pre-selection is similar but multi-trait phenotyping and genotyping is done only one a subset of the population pre-selected for its own performance on one trait (usually growth). **(E)** Under BLUP with sib selection, unselected sibs of the candidates are submitted to a lethal challenge (e.g., disease, processing yields) and genotyped and family values are incorporated in breeding value evaluation for the lethal traits. **(F)** BLUP with sib and pre-selection combines panels **(D,E)**.

Since this date, public organizations and breeding companies initiated selection programs using parentage assignment in sturgeons (France, USA), Atlantic salmon (Ireland, Norway, Scotland), tilapia (Philippines), halibut (Norway and Scotland), rainbow trout (France), cod (Norway), gilthead sea bream (France, Greece, Spain), turbot (France), European sea bass (France, Greece), meagre and red drum (France), Asian sea bass (Singapore, Indonesia, Australia), and shrimps (Australia, Thailand, Mexico, Equator, Central and South America). This list may be incomplete and represents the present informal expert knowledge of the authors. Little information is publicly available in these programs but mass selection, family-based selection (often BLUP: best linear unbiased prediction) or a combination of both are used to improve growth, processing yields, quality traits and disease resistance according to different schemes (Figure 1).

Key parameters to choose to develop a breeding program using parentage assignment are not only the genotyping cost (12–20 Euros per individual), but also the capacity to produce a large number of families in one batch to avoid tank effects, the true assignment efficiency, as well as the availability of tools such as rapid mass genotyping capacities (specially for species with short generation interval), individual tagging to improve traceability and facilitate data collection, automated database systems to collect, store and link performances to tags, DNA samples and pedigrees, optimized genetic softwares to rank and mate candidates to maximize genetic progress and minimize inbreeding. Use of parentage assignment is not only “genetic tagging,” but requires a complete re-optimization of breeding programs.

#### Optimization of breeding schemes using parentage assignment

One main target for optimization has been the limitation of numbers genotyped, using two-way nested models for partial pedigrees (Li et al., 2003), or extreme phenotypes with family effect considered as a fixed effect (Morton and Howarth, 2005). BLUP selection normally requires the knowledge of performance and pedigree on all candidates, which is not the case in Figures 1D,F. In these cases, the loss of selection efficiency (compared to BLUP with pedigree known on all candidates) depends on selection intensity and genetic parameters (Chapuis et al., 2010; Dupont-Nivet et al., 2010b; Sonesson et al., 2011). In addition, issues linked to mixing of families were studied, such as methods to limit non-genetic maternal effect in salmonids (Haffray et al., 2012b), effect of grading practices to limit cannibalism on family contributions in barramundi (Loughnan et al., 2013), and the importance to consider male maturation status to estimate heritability of growth more accurately (Dupont-Nivet et al., 2010a). Ninh et al. (2011) and Sae-Lim et al. (2013) compared expected genetic gains with different systems of evaluation (mixed/separate families and impact of G × E interactions), while Haffray et al. (2013) proposed application of ultrasound tomography to predict processing yields on live candidates to limit the use of slaughtered sibs.

## GLOBAL APPRAISAL AND PERSPECTIVES

The rapid increase of publications using parentage assignment in the last decade shows how powerful this method is to estimate genetic parameters in any species and rearing system. It avoids the initial investment in separate family rearing units and limits associated biases, even more in species with high larval mortality, small larval size, and initial live feeding. Applications are strongly driven by reproductive constraints linked to the need to simultaneously produce enough families (Table 1). The cost/information ratio has to be maximized with adequate management of variance sources (number of parents, initial representation of families, or groups of spawns), mating design, and number of individuals genotyped.

**Table 1:** SWOT analysis of parentage assignment with genomic markers for aquaculture breeding.

**Strengths**	**Weaknesses**
– Absence of common environment effects – Allows any type of mating design – No investment in structures and limited fish rearing costs (labor, consumables) – Allows family evaluation in industry conditions – Microsatellites available in most species – High flexibility	– Each new trait measured on sibs may require additional genotyping to balance with the benefit expected – Biased BLUP estimates if pre-selection done – Unit cost of genotyping sometimes dissuasive – Ability to produce high numbers of families simultaneously needed for full benefits
**Opportunities**	**Threats**
– Ease to develop SNP or microsatellites at low cost in any species with next-generation sequencing technologies – Future use of (within family) genomic selection will decrease the cost of pedigree information and provide within-family relatedness estimates improving accuracy – Many research laboratories with appropriate knowledge in genotyping to initiate programs	– Lack of maintenance of microsatellite genotyping equipment with the advent of SNPs – Limited number of professional genotyping companies high volume, service and automation capacities – Lack of efficiency when maker set assignment power overestimated or genotyping errors too numerous

Optimal investment in parentage assignment is a balance between the reduction of investment and operational costs needed for the separate family rearing and the cost of genotyping, which presently limits the application of parentage assignment to mass selection and family-based selection on a limited number of traits. Moreover, any new trait that cannot be recorded on the live candidate and has to be measured on sibs then requires additional genotyping with a cost/benefit ratio to estimate case by case, and to compare with the possible use of indirect criteria.

A major benefit of parentage assignment is that it allows high selection pressure (<3%) to be applied in commercial conditions, while still controlling inbreeding. The knowledge of pedigree also allows an increase in selection accuracy (and then a higher selection gain) on all traits, as well as selection on lethal traits which cannot be done by individual selection. This technology also allows to easily combine sanitary protection of the breeding nucleus and sib testing in commercial environments. Parentage assignment offers simplicity and flexibility in the life of the breeding program that can be easily adapted to new traits, new mating schemes, different number of candidates. This is critical, especially at the initiation of domestication, for “niche” species or in developing countries, where the need for separate rearing system has often prevented any investment in selective breeding in the past, or has fixed the architecture of the breeding programs.

### Conflict of Interest Statement

The authors declare that the research was conducted in the absence of any commercial or financial relationships that could be construed as a potential conflict of interest.
